# Cloning and Molecular Characterization of *Hsp* Genes from *Anoplophora glabripennis* and Their Responses to Cold Stress

**DOI:** 10.3390/ijms231911958

**Published:** 2022-10-08

**Authors:** Yabei Xu, Yurong Li, Fengming Shi, Sainan Zhang, Shixiang Zong, Jing Tao

**Affiliations:** Beijing Key Laboratory for Forest Pest Control, Beijing Forestry University, Beijing 100083, China

**Keywords:** *Anoplophora glabripennis*, heat shock protein, cold stress, prokaryotic expression, Western blot

## Abstract

*Anoplophora glabripennis* (*Agla*) is an important global quarantine pest due to its highly destructive impacts on forests. It is widely distributed in many countries in Asia, Europe, and North America. The survival of *A. glabripennis* larvae has been facilitated by its high adaptability to low temperature. When insects are subjected to temperature stress, heat shock proteins (Hsps) limit cell damage and improve cell tolerance via their protein folding, localization, and degradation activities. However, the temperature adaptation mechanisms of *A. glabripennis* Hsps remain unclear. In this study, four *A. glabripennis Hsp* genes, *AglaHsp20.43*, *AglaHsp71.18*, *AglaHsp82.09*, and *AglaHsp89.76*, were cloned. Sequence analysis showed that all four Hsps had specific conserved domains. Phylogenetic analysis revealed that Hsps from different subfamilies were evolutionarily conserved, and that AglaHsps were highly similar to those of Coleoptera species. Protein expression vectors (pET30a-*AglaHsps*) were constructed and used to express AglaHsps in *E. coli*, where all four proteins were expressed in inclusion bodies. Western blot analysis showed that AglaHsps were expressed at a range of temperatures, from −10 °C to 25 °C. AglaHsp82.09 and AglaHsp89.76 showed high expressions with treatment at 0 °C. Our results will facilitate clarification of the molecular mechanisms underlying *A. glabripennis* responses to environmental stress.

## 1. Introduction

Heat shock proteins (Hsps) are a group of highly conserved proteins that mitigate the effects of fluctuating environmental conditions by maintaining intracellular protein homeostasis [1,2,3]. Hsps are found in almost all types of cells and tissues in multicellular organisms [4]. Constitutively expressed Hsps are involved in protein folding, intracellular transport, regulation of signal transduction pathways, and cytoskeleton assembly, all of which are essential for normal growth and development [5]. Upon exposure to harsh environmental conditions, such as excessive cold or heat, starvation, or hypoxia, Hsps protect cells from damage by reducing protein misfolding and aggregation, and by assisting in the refolding or degradation of denatured proteins [6].

Insect Hsps are classified as either ATP-independent small heat shock proteins (sHsps) or as larger ATP-dependent proteins, depending on their molecular mass and sequence homology [7]. The sHsp group contains the most widespread and diverse class of molecular chaperones, and these act as the first line of cell defense by preventing non-native protein aggregation [8,9]. Larger ATP-dependent Hsps include Hsp60, Hsp70, and Hsp90. Hsp60, also known as chaperonin, is a large cylindrical oligomer composed of two back-to-back rings. Denatured proteins are encapsulated in the central cavity of the ring and are refolded to their native state in an ATP-dependent manner [10]. Hsp70 performs a wide array of cellular functions, all of which derive from the ability of the N-terminal nucleotide-binding domain (NBD) to allosterically regulate the substrate affinity of the C-terminal substrate-binding domain in a nucleotide-dependent mechanism [11,12]. Hsp70 activity is regulated by Hsp40/J-domain proteins and nucleotide exchange factors (NEFs). When stress is relieved and ATP becomes more plentiful, Hsp40 can then bind and transfer the substrate to Hsp70 for renaturation or degradation. The NEFs induce nucleotide exchange, further accelerating the ATPase cycle and substrate release [7,13,14]. Hsp-organizing protein (HOP) can facilitate the delivery of substrate proteins from the Hsp70/Hsp40 complex to Hsp90 for refolding. HOP binds to the C-terminal MEEVD sequence of both Hsp90 and Hsp70 via separate tetratricopeptide repeat (TPR) domains, and this connection between the two chaperone machineries is critical for cell viability [15,16]. Different Hsps play distinct roles in regulatory networks according to their relative abundance and ATP availability, and act to maintain protein homeostasis in collaboration with cochaperone partners. At present, *Hsp* genes have been cloned and characterized in several insect species, and the relationships between Hsps and temperature adaptability have been determined. For example, the expressions of two *Bthsp70* genes were induced upon exposure to temperature stress in *Bemisia tabaci* pupae and adults. The mortality was significantly higher in *B. tabaci* adults that ingested *dsBthsp70* after exposure at 43 °C for 1 h [17]. Li et al. characterized and cloned six *Hsp70* genes in *Monochamus alternatus* and found that expression of *MaltHsp70-1* and *MaltHsp70-2* increased 2904- and 8898-fold, respectively, after treatment at 45 °C for 3 h. In addition, survival of heat-stressed *E. coli* increased by 6.17% (to 12.17%) upon transformation with PET-28a-Hsp70 [18]. Expression of *sHsps* in *Chilo suppressalis* and *Liriomyza trifolii* was upregulated after high-temperature treatments [19,20], further supporting the wide involvement of Hsps in insect responses to temperature stress. 

The Asian long-horned beetle *Anoplophora glabripennis* (Coleoptera: Cerambycidae) is a polyphagous wood-boring pest native to Asia that primarily damages broad-leaved trees such as *Populus*, *Salix*, *Ulmus*, and *Acer*. *A. glabripennis* adults feed on twigs, petioles, and leaves of host plants for complementary nutrition. Larvae mainly damage the phloem and xylem of trees, destroying the vascular tissue and wood structure, leading to the decline of forest productivity and even tree death [21]. The natural dispersal ability of the *A. glabripennis* adult is limited [22,23]; however, global trade has facilitated its dispersal to the world via wooden packaging materials, posing a serious threat to local urban trees [24]. Since the initial report of invasive populations in the USA in 1996, local outbreaks have been observed in Austria (2001), Canada (2003), France (2003), Germany (2004), and Italy (2007) [25,26]. *A. glabripennis* is currently listed as a critical quarantine pest in Europe and North America [27,28]. In China, *A. glabripennis* produces one generation every 1–2 years and overwinters as larvae, eggs, or pupae [29]. After the launch of the “Three North” Shelter Forest Program in the 1980s, *A. glabripennis* became widespread in most parts of China [30], and in recent years has been found at higher latitudes and altitudes, and in colder regions, such as Heilongjiang, Qinghai, and Tibet [31]. Insects are poikilotherms, and adaptation to low temperatures is thus an important factor for the successful colonization of *A. glabripennis* in novel dispersal areas. *A. glabripennis* larvae were found to produce cryoprotectants such as glycerol, glucose, and free amino acids during overwintering, leading to improved cold tolerance [32]. Transcriptome analysis of *A. glabripennis* larvae at different overwintering stages showed that the expression of heat shock protein, glycerol kinase, aquaporin, and ribosomal protein genes initially increased and then decreased with the changes of ambient temperature [33]. In total, 47 *Hsp* genes were identified in the *A. glabripennis* genome, 13 of which were significantly upregulated during overwintering. Analysis by qPCR showed that *Hsp* gene expression responded to low-temperature stress [34], but protein expression patterns were not elucidated.

In this study, four *A. glabripennis Hsp* (*AglaHsp*) genes were cloned, and their sequence characteristics and phylogenetic relationships were analyzed. Prokaryotic expression vectors for the four AglaHsps were constructed and expressed in *E. coli*. The expression patterns under low-temperature stress were assessed using Western blotting analysis. These results provide a theoretical basis for clarifying the biological function of Hsps under abiotic stress and provide a basis for elucidating the molecular mechanisms underlying the response to temperature stress during the dispersal and colonization of *A. glabripennis*.

## 2. Results

### 2.1. Cloning and Sequence Analysis of AglaHsps

The complete coding sequences of four *AglaHsp* genes were cloned and denoted as *AglaHsp20.43* (GenBank accession number: OP131592), *AglaHsp71.18* (GenBank accession number: OP131593), *AglaHsp82.09* (GenBank accession number: OP131594), and *AglaHsp89.76* (GenBank accession number: OP131595) according to the molecular weights of their predicted proteins. The coding sequences and their deduced amino acid sequences are shown in Figure S1. AglaHsp20.43 belonged to the sHsp family and encoded 181 amino acids, including a predicted α-crystallin domain (ACD) at positions 56–165. The instability coefficient of AglaHsp20.43 was 44.10, indicative of an unstable protein. AglaHsp71.18 encoded 649 amino acids and contained a predicted Hsp70 heat shock domain at positions 3–649. The predicted protein sequence also contained three Hsp70 signature sequences (IDLGTTYS, IFDLGGGTFDVSIL, VVLVGGSTRIPKIQQ) and a conserved cytoplasmic localization motif (EEVD) at the extreme C-terminus. AglaHsp82.09 and AglaHsp89.76 respectively belonged to the Hsp90A and Hsp90B families and encoded 717 and 784 amino acids. AglaHsp82.09 contained a Hsp90 heat shock domain and a Hsp90 signature sequence (YSNKEIFLRE) at positions 11–717 and 31–40, respectively, as well as a C-terminal EEVD motif indicative of cytoplasmic localization. AglaHsp89.76 had a Hsp90 heat shock domain at positions 257–776 and a Hsp90 signature sequence (YRNKEIFLRE) at positions 95–104. AglaHsp89.76 also exhibited an endoplasmic reticulum localization motif (HDEL) at the C-terminal and a 20-residue N-terminal signal peptide indicative of a secreted protein. All four Hsps were predicted to be hydrophilic proteins with no transmembrane structures. Full physicochemical properties, including isoelectric point and instability index, are listed in Table 1.

**Figure 1:**
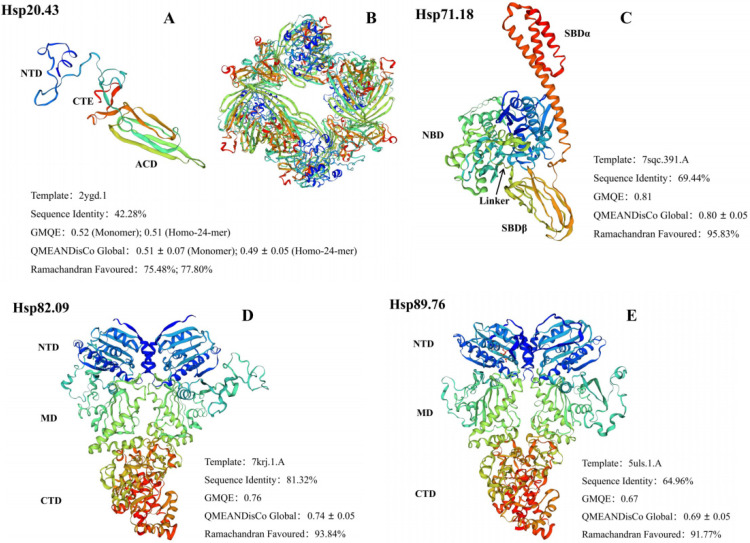
Three-dimensional structures of AglaHsps proteins. *Homo sapiens* α-Crystallin protein (PDB ID: 2ygd), *Chlamydomonas reinhardtii* Hsp70A (PDB ID: 7sqc), *Homo sapiens* Hsp90-alpha (PDB ID: 7krj), and *Canis familiaris* Endoplasmin (PDB ID: 5uls) were used as templates to predict the 3D structures of AglaHsp20.43 (**A**,**B**), AglaHsp71.18 (**C**), AglaHsp82.09 (**D**), and AglaHsp89.76 (**E**), respectively.

The secondary structures of the four AglaHsps contained α-helix, β-sheet, β-turn, and random coil (Table 2). *Chlamydomonas reinhardtii* Hsp70A (PDB ID: 7sqc), *Homo sapiens* Hsp90-alpha (PDB ID: 7krj), and *Canis familiaris* Endoplasmin (PDB ID: 5uls) were used as best modeling templates for AglaHsp71.18, AglaHsp82.09, and AglaHsp89.76, respectively. The 3D structure of AglaHsp71.18 revealed the existence of an N-terminal NBD, a C-terminal substrate-binding domain (SBDβ), and a helical lid domain (SBDα) (Figure. 1C). The position of the linker in *E. coli* DnaK (^388^DVLLLD^393^) suggested that the corresponding ^388^DVLLVD^393^ sequence in AglaHsp71.18 might act as a linker to connect NBD and SBDβ, and that the highly conserved hydrophobic linker might have a key role in NBD function and allostery. Hsp90 exists as a dimer, and dimerization is essential for its function in vivo. The 3D structures of AglaHsp82.09 and AglaHsp89.76 indicated that each monomer had a highly conserved N-terminal domain (NTD) connected to a middle domain (MD), followed by a C-terminal domain (CTD) (Figure 1D,E). Hsp90 binds ATP in its NTD, which is connected to the middle domain by a charged linker that is highly variable in length and composition between species. The 3D model of AglaHsp20.43 predicted using a *Homo sapiens* α-crystallin protein (PDB ID: 2ygd) was poor quality. AglaHsp20.43 was predicted as an oligomer of 24 subunits, with each monomer containing a variable N-terminal domain, a more conserved β-sandwich α-crystallin domain, and a C-terminal extension (Figure 1A,B). A Ramachandran plot showed that only 75.48% and 77.80% of the AglaHsp20.43 monomer and oligomer residues, respectively, were in most favored regions. Therefore, electron microscopy, X-ray, or other techniques are needed to determine a more accurate 3D structure for AglaHsp20.43.

### 2.2. Multiple Sequence Alignment and Phylogenetic Analysis of AglaHsps

**Figure 2:**
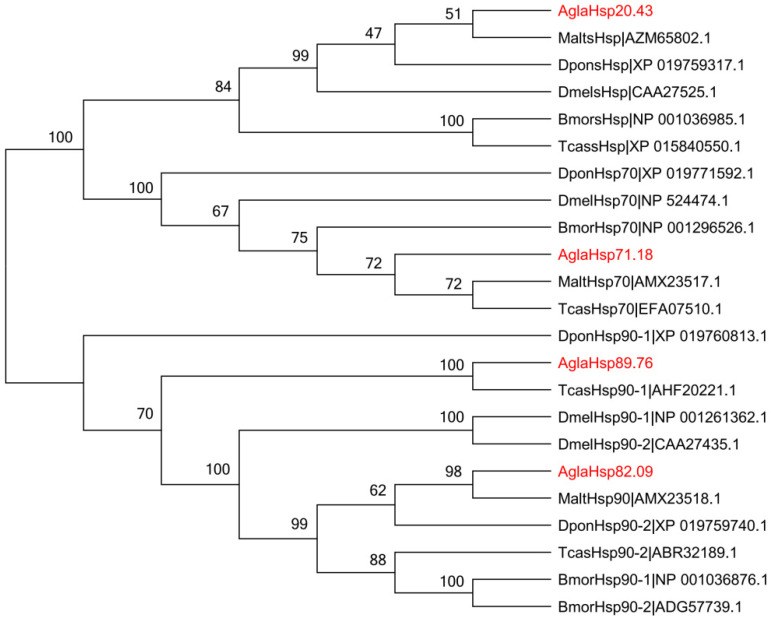
Phylogenetic analysis of Hsps from *Anoplophora glabripennis*, *Tribolium castaneum*, *Monochamus alternatus*, *Dendroctonus ponderosae*, *Drosophila melanogaster*, and *Bombyx mori*. An unrooted phylogenetic tree of Hsps from six insect species was constructed using MEGA6.05 with the Neighbor-Joining method (node support based on 1000 bootstrap replications is shown). The four AglaHsps are indicated in red.

Hsp sequences from *A. glabripennis*, *Tribolium castaneum*, *Monochamus alternatus*, *Dendroctonus ponderosae*, *Drosophila melanogaster*, and *Bombyx mori* were aligned to construct an unrooted phylogenetic tree. The results showed that Hsps from the same subfamily clustered together (Figure 2), indicating their evolutionary conservation. The four *A. glabripennis* Hsps were highly similar to those of Coleoptera species. AglaHsp20.43 shared 17.65–36.97% similarity with other sHsps, which was most similar to MaltsHsp and clustered in the same evolutionary branch. The similarity of AglaHsp71.18 to MaltHsp70 and TcasHsp70 reached more than 85%. Eleven Hsp90s were divided into cytoplasm, endoplasmic reticulum, and mitochondrial types, and large differences were observed among different Hsp90 types. For example, Cy-AglaHsp82.09 was 76.02–85.54% similar to seven Cy-Hsp90s, but it exhibited similarities to ER-AglaHsp89.76 and ER-TcasHsp90-1 of 40.54% and 40.91%, respectively, and similarity to Mi-Hsp90s of only 26.58%.

### 2.3. Prokaryotic Expression and Purification of AglaHsps

**Figure 3:**
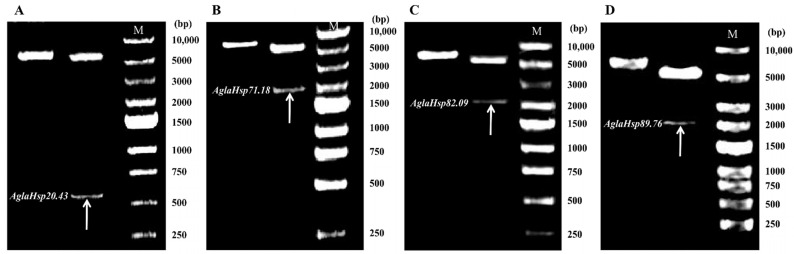
Recombinant plasmids pET30a-*AglaHsps* and their double digestion products. On each panel, the left band represents the intact recombinant plasmid pET30a-*AglaHsps*, and the right band shows the digested products, plasmid pET30a and full-length CDS of *AglaHsp20.43* (**A**), *AglaHsp71.18* (**B**), *AglaHsp82.09* (**C**), and *AglaHsp89.76* (**D**), with their signal peptide regions removed. Target genes are indicated with arrows. M: DNA fragment size marker (DNA Ladder 250–10,000 bp).

**Figure 4:**
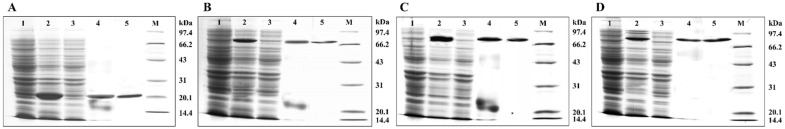
SDS-PAGE analysis of recombinant protein for AglaHsp20.43 (**A**), AglaHsp71.18 (**B**), AglaHsp82.09 (**C**), and AglaHsp89.76 (**D**). Channels 1, 2, 3, 4, and 5 represent the bacterial culture without IPTG induction (1), bacterial culture induced with IPTG (2), the supernatant of bacterial culture after induction (3), the pellet of bacterial culture after induction (4), and purified recombinant protein (5). M: Protein fragment size marker (Protein Ladder 14.4–97.4 kDa).

Construction of recombinant pET30a-*AglaHsps* prokaryotic expression vectors was verified by double digestion to produce two clear electrophoretic bands representing plasmid pET30a and full-length CDS of *AglaHsp20.43*, *AglaHsp71.18*, *AglaHsp82.09*, or *AglaHsp89.76* with their signal peptide regions removed (Figure 3). Protein expression was induced with IPTG, and bacterial culture without induction was used as a control. SDS-PAGE analysis of IPTG-induced culture showed produced proteins of approximately 20 kDa (Hsp20.43), 70 kDa (Hsp71.18), 80 kDa (Hsp82.09), and 90 kDa (Hsp89.76), indicating the successful expression of recombinant proteins (Figure 4). Supernatant and pellet fractions obtained after ultrasonication and centrifugation of induced bacterial cultures were also assessed by SDS-PAGE. All four Hsps were found in the bacterial pellet, with no clear expression in the supernatant, showing that the recombinant proteins were expressed as inclusion bodies. Ni-Column was used to purify the recombinant proteins. After protein renaturation, a single target protein of the expected size was observed with SDS-PAGE analysis.

### 2.4. Antibody Titer Determination and Western Blotting Analysis

**Figure 5:**
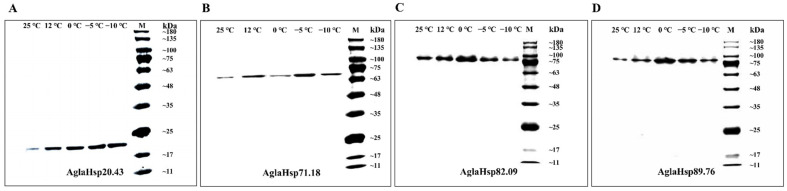
Western blot analysis of AglaHsp20.43 (**A**), AglaHsp71.18 (**B**), AglaHsp82.09 (**C**), and AglaHsp89.76 (**D**) expressions after treatment with different temperatures. Expression levels of the target proteins were estimated according to the band intensities. M: Protein fragment size marker (Protein Ladder 11–180 kDa).

ELISA analysis indicated that the anti-AglaHsps polyclonal antibody titers were 1:1,280,000, 1:1,280,000, 1:1,280,000, and 1:320,000 for AglaHsp20.43, AglaHsp71.18, AglaHsp82.09, and AglaHsp89.76, respectively. AglaHsps expression at the protein level was examined after instantaneous cooling for 6 h at 25 °C, 12 °C, 0 °C, −5 °C, or −10 °C. Western blot analysis of protein extracts from *A. glabripennis* larvae showed the single non-dispersive immune bands in the corresponding positions, indicating the expressions of the four proteins under all treatments (Figure 5). According to the immunoreactive band intensity, the expressions of AglaHsp20.43 under low-temperature treatments were higher than that of the control, but with no significant difference between different treatments. The AglaHsp71.18 expressions increased except treatment at 0 °C. The expression levels of AglaHsp82.09 and AglaHsp89.76 at 0 °C were significantly higher than other groups. This further illustrated that AglaHsps were involved in the response of *A. glabripennis* larvae to low-temperature adaptation.

## 3. Discussion

Temperature is an important factor affecting insect abundance and distribution. Extreme heat and cold disrupt protein folding, causing protein denaturation and aggregation, and leading to cell death [5,7]. Insects respond to environmental stress through complex defense systems, which are closely related to biochemical and molecular regulation. Insects can improve cold tolerance by reducing their supercooling point and regulating water content, as well as accumulating glycerol, polyols, sugars, and other cryoprotectants [35]. Gene expression change is also a major component of various stress responses. Previous studies showed that environmental temperature fluctuations can induce the adaptive expression of insect *Hsp* genes. Huang et al. identified and cloned three *sHsp* genes in *Liriomyza sativa* that exhibited different sensitivities to cold [36]. *Eurosta solidaginis* larvae showed consistent elevation of *Hsp110*, *Hsp70*, *Hsp40*, *Grp78*, and *sHsp* expression over the late autumn and winter months, generally at levels 1.5–2.0-fold higher than September expressions, suggesting that these proteins contributed to cell survival over the winter via protection and stabilization of macromolecules [37].

### 3.1. The Diversified Structures of AglaHsps Underlie Their Functional Specificities

In this study, four *A. glabripennis Hsp* genes, *AglaHsp20.43*, *AglaHsp71.18*, *AglaHsp82.09*, and *AglaHsp89.76*, were cloned, and the proteins were expressed in *E. coli*. AglaHsp20.43 included a conserved ACD with a β-sandwich structure. Different subunits form dimers through the interaction of β-strands in the ACD, and these constitute the basic structural unit of sHsps [8,38,39]. Monomers of sHsp vary in size, at 12–43 kDa [40], and the majority of sHsps assemble into large oligomers comprising a minimum of 12 monomers. *Triticum aestivum* Hsp16.9 and *Arabidopsis* Hsp21 form dodecamers; *Methanocaldococcus jannaschii* Hsp16.5 and *Saccharomyces cerevisiae* Hsp26 complexes have 24 subunits [8,41,42]. Three-dimensional structure prediction indicated that AglaHsp20.43 consisted of 24 subunits. Each sHsp subunit binds to a substrate protein of almost equal molecular weight without the input of ATP, and sHsps may thus be the main contributor to the chaperone capacity of cells [38,39,41]. Furthermore, the plasticity of sHsp quaternary structure may be functionally important for recognizing and binding a diverse population of conformationally flexible target proteins [43]. A conserved cytoplasmic localization motif, EEVD (Glu-Glu-Val-Asp), was identified at the extreme C-terminus of AglaHsp71.18 and AglaHsp82.09, which mediates interactions with TPR domains of the HOP, thereby facilitating substrates handover from Hsp70 to Hsp90 [44,45,46]. The Hsp70 linker is a central component of the mechanism that couples ATP binding and hydrolysis to conformational changes in the substrate-binding domain. The linker, which consists of a stretch of four hydrophobic residues (mostly leucine) flanked by two aspartic acid residues, is highly conserved within the Hsp70 subfamily [47]. Our analysis indicated that residues 388–393 (DVLLVD) of AglaHsp71.18 acted as a linker to connect NBD with SBDβ. Binding of ATP to NBD triggers the attachment of the linker and the α-helical lid of SBD to the NBD, opening the peptide-binding pocket [48]. The most-C-terminal motif (KDEL) of endoplasmic reticulum Hsp90 (ER-Hsp90) was previously thought to be a signature sequence of the subfamily [49,50], but it was recently shown to be not completely conserved among ER-Hsp90 members. Chen et al. analyzed all Hsp90 genes available in 32 complete genomes and found that 38.7% of the sequences in the alignment had H, A, E, R, S, or N residues at the K site [51]. AglaHsp89.76 contained a 20-residue N-terminal signal peptide and a C-terminal HDEL motif. Combined with its predicted subcellular localization, these observations suggest that AglaHsp89.76 might play a role in the quality control of endoplasmic reticulum proteins. 

### 3.2. Evolutionary Trends and Environmental Adaptabilities of AglaHsps

Comparative and homology analysis of *Hsp* gene families in different species showed that all the organisms studied contained virtually the same classes of *Hsp* genes, which exhibited high similarity even between phylogenetically distant organisms. This suggested that the appearance of *Hsp* genes in eukaryotes predated their species divergence [5]. As a class of diversified molecular chaperones, the number of sHsps varies greatly across different organisms: 19 in *Arabidopsis thaliana*, 4 in *Drosophila melanogaster*, and only 2 in *Saccharomyces cerevisiae* [8,41,42]. The sHsp family also exhibits extensive sequence variation and evolutionary differences relative to Hsp70 and Hsp90 molecular chaperones [52]. In this study, the similarity between AglaHsp20.43 and the other five sHsps was only 17.65–36.97%. Despite the high sequence variability, the α-crystallin domain is an evolutionarily conserved hallmark of the sHsp family [9]. Work conducted by Thomas on cytochrome P450 in vertebrates indicated that phylogenetically stable genes tend to have conserved core functions, while phylogenetically diverse genes are much more varied in their function or, possibly, in their substrate specificities [41,53]. However, little is known regarding whether sHsp members have similar relationships with substrates. Hsp70 proteins and their constitutive cognates Hsc70 are highly evolutionarily conserved [12]. Eukaryotic Hsp70s are widely distributed in the cytoplasm, endoplasmic reticulum, mitochondria, and chloroplasts, and share approximately 50% sequence identity with prokaryotic *E. coli* DnaK [13]. In this study, the similarity between AglaHsp71.18 and five Hsp70 proteins was more than 75%, and the similarity of AglaHsp71.18 with MaltHsp70 and TcasHsp70 was 86.68% and 87.14%, respectively. In some cases (such as the *GRP78*-like genes), sequence similarities are greater among genes from different species, revealing the early gene duplication events [2]. Conservation of Hsp70 is also reflected by the conserved cross-species functions [54]. For example, overexpression of *Drosophila* Hsp70 in monkey COS cells produced a more rapid recovery of nucleolar morphology after heat shock [55]. When transgenic mice containing the porcine *Hsp70* were exposed to heat stress, Hsp70 overexpression reduced stress-induced hypothalamic damage, and hypothalamic nerve samples from surviving mice exhibited improved adaptation [56]. The Hsp90 family can be divided into five subfamilies: cytosolic Hsp90A, endoplasmic reticulum (ER)-localized Hsp90B, chloroplast Hsp90C, mitochondrial TNFR-associated protein (TRAP), and bacterial high-temperature protein G (HtpG) [51]. Of these, cytosolic Hsp90A is the largest and most widespread of the Hsp90 families [57] and shares 50% sequence similarity with the ER-localized Hsp90B (known as GRP94 in humans). HtpG, which is found in most bacteria, shares 42% sequence similarity with human Hsp90. Despite this, HtpG is not essential in non-stressful conditions and has only modest effects on growth at high temperatures [58]. The sequence similarities of 11 insect Hsp90s that localized to the cytoplasm, ER, and mitochondria were assessed. The results showed that Hsp90s from the same subfamily exhibited high similarities and were divided into the same evolutionary group. Structurally, Hsp90A lacks signal peptides at the N-terminus, and 11 conserved motifs distinguish the proteins from other subfamily members. Signal peptides in Hsp90B and TRAP proteins facilitate their export into the ER and mitochondria to function, respectively [51]. Overall, Hsps form unique protein structures through different evolutionary trends to provide optimal capacity for organisms to respond to environmental fluctuations.

### 3.3. AglaHsps Function in the Regulatory Networks

Gene expression regulation plays a key role in the process of biological adaptation to environmental stress. The regulation of adaptive responses may be realized at the levels of transcription induction, translation efficacy, and/or stability of resulted mRNAs or proteins. Large-scale transcriptomic analysis in a wide range of species showed that up to 40% of genes exhibited altered expression patterns after exposure to temperature stress [5]. The temperature-responsive system includes RNA- and DNA-modifying enzymes, carbohydrate metabolism enzymes, antioxidant proteins, and regulatory proteins such as transcriptional factors and protein kinases [59,60,61,62,63]. Regulation of stress-related genes at the transcriptional and translational levels depends on the severity and duration of stress applied [64]. Previous qPCR analysis showed that *AglaHsp20.43*, *AglaHsp71.18*, *AglaHsp82.09*, and *AglaHsp89.76* could be induced by low temperatures but with different expression patterns [34]. After instantaneous cooling treatments at 0 °C for 6 h, the expressions of *AglaHsp82.09* and *AglaHsp89.76* were 5.15- and 5.07-fold higher than that of controls, respectively, but further decreased with additional decreases in temperature. *AglaHsp20.43* and *AglaHsp71.18* expressions under different low-temperature treatments were increased but withoot a significant difference compared with the control group (Figure S2). In this study, four Hsp recombinant proteins were successfully expressed in *E. coli* BL21DE3 competent cells. Western blot analysis showed that AglaHsps were expressed at a range of temperatures. The expression of AglaHsp82.09 and AglaHsp89.76 was the highest at 0 °C, while AglaHsp71.18 expression at 0 °C had no significant change compared with the control. Previous research indicated that Hsps are abundant within cells during or after stress, and their synthesis, maintenance, and degradation consume substantial energy and amino acid resources and also occupy the protein synthesis machinery, thus causing cellular damage [65,66]. Thus, expression of Hsps in each species and geographic population is a balance between benefits (high temperature tolerance) and costs (negative effects of Hsps overexpression on growth, development, and other characteristics). This may explain why Hsp expressions decreased with further decreases in temperature below 0 °C. In fact, the increased synthesis of a single Hsp does not sufficiently explain the temperature tolerance of a whole cell and organism. This study examined expression levels of only four Hsps, but 47 *Hsp* genes have been identified in the genome of *A. glabripennis*, and each Hsp likely plays a specific role in protecting cells from stress. It is therefore important to clarify the role of Hsp molecular regulatory networks in the temperature tolerance of *A. glabripennis* larvae. If the loss of a certain Hsp gene was found to be nonlethal, gene knockout or RNA interference (RNAi) could be used to elucidate its function and further to reveal the regulatory mechanisms of temperature adaptation in *A. glabripennis* larvae.

## 4. Materials and Methods

### 4.1. Insect Collection and Temperature Treatments

*A. glabripennis* larvae were collected from a natural poplar forest in Yanchi county, Ningxia Hui Autonomous Region, China (37°47′ N, 107°23′ E). Larvae were isolated from xylem and transported to the laboratory in Beijing within 48 h. Fourth or fifth instar larvae (the predominant overwintering instar of *A. glabripennis* larvae) displaying good growth were selected by measuring head shell width according to the He et al. method for temperature treatments [67]. A total of 25 larvae were used in the instantaneous cooling treatments with five biological replicates per group. Larvae were treated at 25 °C, 12 °C, 0 °C, −5 °C, or −10 °C for 6 h, respectively. Treated samples were immediately frozen in liquid nitrogen and stored at −80 °C for subsequent RNA extraction and analysis. 

### 4.2. RNA Extraction and cDNA Library Construction

Total RNA was extracted from larvae using the TRIzol reagent (No. 15596026; Invitrogen, Carlsbad, CA, USA) and the RNeasy Plus Mini Kit (No. 74134; Qiagen, Hilden, Germany) according to the manufacturers’ instructions. RNA purity, concentration, and integrity were assessed using the NanoDrop 8000 spectrophotometer (Thermo, Waltham, MA, USA) and agarose gel electrophoresis. cDNA libraries were constructed from 1 µg of total RNA using the PrimeScript RT Reagent Kit with gDNA Eraser (No. RR047A; TaKaRa, Dalian, China) and were stored at −20 °C for subsequent analysis.

### 4.3. Cloning of AglaHsp Genes

Four candidate *Hsp* genes were selected based on the larval overwintering transcriptomes (PRJNA613658) and the *A. glabripennis Hsp* gene family analysis [33,34]. The open reading frame (ORF) regions of candidate genes were predicted using ORF finder (https://www.ncbi.nlm.nih.gov/orffinder, accessed on 20 June 2021), and their integrity was confirmed using BLAST with sequence alignment. Specific primers for amplification of full-length sequences were designed using Primer Premier 5.0 (Table S1). PCR conditions are shown in Table S2. Amplicons were verified using 1% agarose gel electrophoresis, purified, and cloned into the pEASY-Blunt Simple Cloning Vector (No. CB111-01; TransGen Biotech, Beijing, China). Ligated products were then transformed into Trans1-T1 Phage-Resistant Chemically Competent Cells (No. CD501-01; TransGen Biotech, Beijing, China). Positive transformants were selected using blue-white spot screening and sequenced at RuiBiotech Co., Ltd. (Beijing, China) to verify the inserted target coding sequences (CDS).

### 4.4. Bioinformatic Analysis

Cloned nucleic acid sequences were translated into amino acid sequences using the ExPASy Translate tool (https://web.expasy.org/translate/, accessed on 10 April 2022). ProtParam software (https://web.expasy.org/protparam/, accessed on 10 April 2022) was used to obtain the amino acid composition, molecular weight, isoelectric point, and instability index of the AglaHsps proteins. The candidate genes were denoted as *AglaHsp* and identified by their molecular weight. Amino acid sequences were analyzed for hydrophilicity, hydrophobicity, and transmembrane structures using ProtScale (https://web.expasy.org/protscale/, accessed on 10 April 2022) and TMHMM 2.0 (https://services.healthtech.dtu.dk/service.php?TMHMM-2.0, accessed on 10 April 2022). Subcellular localization and signal peptides were predicted using WoLF PSORT (https://wolfpsort.hgc.jp/, accessed on 11 April 2022) and SignalP 6.0 (https://services.healthtech.dtu.dk/service.php?SignalP, accessed on 11 April 2022). The NCBI Conserved Domain Database (https://www.ncbi.nlm.nih.gov/Structure/cdd/wrpsb.cgi, accessed on 11 April 2022) and PROSITE (https://prosite.expasy.org/, accessed on 11 April 2022) were used to confirm conserved domains. Secondary structure and three-dimensional models of AglaHsps were predicted using SOPMA (https://npsa-prabi.ibcp.fr/cgi-bin/npsa_automat.pl?page=npsa_sopma.html, accessed on 12 April 2022) and SWISS-MODEL (https://swissmodel.expasy.org/interactive, accessed on 10 May 2022). Homologous sequences of candidate genes were identified using BLASTP. Multiple sequence alignments of Hsps were generated using ClustalW, and an unrooted phylogenetic tree was constructed using MEGA 6.05 with the Neighbor-Joining (NJ) method and the following parameters: Poisson correction, partial deletion (site coverage cutoff = 50%), and bootstrap test with 1000 replications.

### 4.5. Prokaryotic Expression and Protein Purification

PCR primers incorporating restriction enzyme sites and protected bases were designed to amplify full-length *AglaHsp* CDS regions with signal peptide sequences removed (Table S1). Cloned plasmids were used as amplification templates, with PCR conditions as shown in Table S2. Purified PCR products were ligated into cloning vectors and then transformed into competent cells. Positive clones were selected by blue-white spot screening and confirmed by sequencing. Plasmids were extracted using the QuickPure Plasmid Mini kit (No. CW2619; CWBIO), digested with appropriate restriction enzymes, and purified. The purified target gene fragments were ligated into the pET-30a(+) expression vector (No. P3120; Solarbio) using T4 DNA Ligase (No. FL101-01; TransGen Biotech) and transformed into *E. coli* BL21 (DE3) competent cells (No. C1400; Solarbio). Positive clones were confirmed by sequencing. Expression of AglaHsps in cell culture was induced with isopropyl-β-D-thiogalactopyranoside (IPTG) at a final concentration of 1 mM at 37 °C. To obtain expressed AglaHsps, cultures were sonicated and then centrifuged at 12,000× *g* at 4 °C for 15 min. Expression of AglaHsps in the supernatant and inclusion bodies was analyzed by SDS-PAGE electrophoresis. Ni-NTA Resin (No. DP101-01; TransGen Biotech) and Affinity Columns (No. DS0106; Solarbio) were used to purify AglaHsps, and purified proteins were renatured with renaturation solutions containing different urea concentrations (6, 4, 3, 2, 1, 0.5 M) in turn. AglaHsps concentrations were determined using the BCA Protein Assay Kit (No. CW0014; CWBIO).

### 4.6. Preparation of AglaHsps Antibody

Purified AglaHsps (500 µg) was emulsified with an equivalent volume of Freund’s complete adjuvant and injected into the subcutaneous muscle of adult female rabbits for primary immunization. After 14, 28, and 42 days, additional purified AglaHsps (500 µg) emulsified with an equivalent volume of Freund’s incomplete adjuvant was injected as before for reimmunization until the required amount of antibody was produced. Immunized serum was purified to collect antibodies. Antibody concentration was assessed using UV spectrophotometer, and antibody titer was determined by enzyme-linked immunosorbent assay (ELISA).

### 4.7. Western Blotting Analysis

Total protein was extracted from *A. glabripennis* larvae using the Total Protein Extraction Kit for Insects (No. SA-07-IS; Invent Biotechnologies). SDS-PAGE electrophoresis and the BCA Protein Assay Kit (No. CW0014; CWBIO) were used to assess protein integrity and concentration. Quantified protein samples (40 µg) were extracted for SDS-PAGE electrophoresis and then transferred to a polyvinylidene fluoride (PVDF) membrane by transfer membrane electrophoresis. The PVDF membrane was incubated overnight in 5% skim milk, and then incubated first with anti-AglaHsps antibody at a 1:3000 dilution for 2 h, followed by HRP-conjugated goat anti-rabbit IgG-HRP (No. SE134; Solarbio) at a 1:1000 dilution at room temperature for 2 h. The DAB Kit (No. CW0125; CWBIO) was used to detect immunoreactive bands.

## 5. Conclusions

In this study, four *Hsp* genes from sHsp, Hsp70, and Hsp90 subfamilies were successfully cloned. Sequence characterization and phylogenetic analysis showed that all four Hsps contained unique protein domains and were divided into different evolutionary groups. The C-terminal of AglaHsp82.09 and AglaHsp89.76 contained cytoplasmic and endoplasmic reticulum localization motifs and clustered with similar Cy-Hsp90s and ER-Hsp90s, respectively, indicating the evolutionary conservation and functional diversity of Hsp subfamilies. Western blot analysis showed that the four Hsps were expressed at a range of temperatures from −10 °C to 25 °C, suggesting a role for them in the response to low temperatures in *A. glabripennis* larvae. The AglaHsps in this study can therefore be used as molecular targets for gene knockout or RNAi to further clarify the interactions of each Hsp member within the regulatory system.

## Figures and Tables

**Table 1:** Sequence characteristics of AglaHsps.

	AglaHsp20.43	AglaHsp71.18	AglaHsp82.09	AglaHsp89.76
Coding sequence	546 bp	1950 bp	2154 bp	2355 bp
Amino acid residues	181 aa	649 aa	717 aa	784 aa
Molecular weight (kDa)	20.43	71.18	82.09	89.76
Theoretical isoelectric point	6.50	5.52	5.00	4.88
Instability index	44.10	37.03	38.39	40.06
Hydrophilic protein	−0.631	−0.427	−0.665	−0.600
Transmembrane structures	No	No	No	No
Signal peptide	No	No	No	aa 1-20
Subcellular localization	Cytoplasmic	Cytoplasmic	Cytoplasmic	Endoplasmic reticulum

**Table 2:** Secondary structure analysis of AglaHsps.

	Alpha Helix	Extended Strand	Beta Turn	Random Coil
AglaHsp20.43	24.86%	16.02%	5.52%	53.59%
AglaHsp71.18	41.29%	18.80%	7.40%	32.51%
AglaHsp82.09	48.81%	15.76%	5.86%	29.57%
AglaHsp89.76	51.02%	13.78%	3.70%	31.51%

## Data Availability

All data are included in the text and Supplementary Materials.

## Supplementary Materials

The following supporting information can be downloaded at: https://www.mdpi.com/article/10.3390/ijms231911958/s1.
