# Supplementary material for: Cloning and Molecular Characterization of Hsp Genes from Anoplophora glabripennis and Their Responses to Cold Stress
Source: Int J Mol Sci. 2022 Oct 8;23(19):11958. doi: 10.3390/ijms231911958 (PMC9570420; doi:10.3390/ijms231911958)
Supplement: Supplementary file 1 [file ijms-23-11958-s001.zip › Figure S1 The coding sequences and their deduced amino acid sequences for AglaHsp20.43, AglaHsp71.18, AglaHsp82.09, and AglaHsp89.76.pdf]

A

```
1     ATGTCTCTTCTGCCGTATTTGTTAGACGACTCGCACTTTCTAAGACCATCTCGGCTCATAGATCAACATTTTGGTCTGACGTTGGATCCA
1      M  S  L  L  P  Y  L  L  D  D  S  H  F  L  R  P  S  R  L  I  D  Q  H  F  G  L  T  L  D  P
91    AATGATTTTCTTCAACCCTTAACTGTACCCAGGATGGTAATAAAATGTCCGGCAGGATATCTAAGAAATTGGAGGTCCTCTGCGTCTCAA
31     N  D  F  L  Q  P  L  T  V  P  R  M  V  I  K  C  P  A  G  Y  L  R  N  W  R  S  S  A  S  Q
181   CAAGACACCGGATCCACTGTCAAATTGGACAAGGACAAATTCCATGCAAGTTTGGACGTACAGCAGTTCAAACCGGAAGAAATTACCGTA
61     Q  D  T  G  S  T  V  K  L  D  K  D  K  F  H  A  S  L  D  V  Q  Q  F  K  P  E  E  I  T  V
271   AAAGTTACTGGAGAAAATACAATTACAGTGGAGGGTAAGCACGAGGAGAAAGAGGATGAGCACGGCCATATTTACAGACATTTTGTCAGG
91     K  V  T  G  E  N  T  I  T  V  E  G  K  H  E  E  K  E  D  E  H  G  H  I  Y  R  H  F  V  R
361   AAATACGTTTTGCCCAAGAATTGCGATATCAATAAGGTAGAGTCCAAGTTGTCTTCTGATGGAGTTTTAACTATTGCCGCTCCGAAGATT
121    K  Y  V  L  P  K  N  C  D  I  N  K  V  E  S  K  L  S  S  D  G  V  L  T  I  A  A  P  K  I
451   GGAGGCAATGAAATCGAACATAAAGAAGTTCCCATTACCCATACTGGAGAGCCAGTCAAGGCTTTGGAACAACCAAAAGCAGGAGATAAA
151    G  G  N  E  I  E  H  K  E  V  P  I  T  H  T  G  E  P  V  K  A  L  E  Q  P  K  A  G  D  K
541   AAGTAA
181    K  *
```

B

```
1     ATGGTTGCAGTTGGTATCGACTTGGGAACAACCTACTCCTGTGTAGGCGTGTGGCAGCATGGAAAAGTGGAAATCATCGCCAACGATCAA
1      M  V  A  V  G  I  D  L  G  T  T  Y  S  C  V  G  V  W  Q  H  G  K  V  E  I  I  A  N  D  Q
91    GGCAACAGGACGACGCCGAGTTATGTTGCATTTACGGACACCGAGCGTCTCTTGGGAGACGCCGCCAAGAACCAGGTCGCCATGAACCCC
31     G  N  R  T  T  P  S  Y  V  A  F  T  D  T  E  R  L  L  G  D  A  A  K  N  Q  V  A  M  N  P
181   AGTAACACCGTCTTCGACGCTAAGAGATTGATCGGCCGCAAGTATGACGACTCGAAGATCCAGCAGGACCTCAAACATTGGCCTTTTAAA
61     S  N  T  V  F  D  A  K  R  L  I  G  R  K  Y  D  D  S  K  I  Q  Q  D  L  K  H  W  P  F  K
271   GTTGTGGACGACTGCGGGAAGCCCAAGATACAAGTAATGTACAAGGGTGAAAAGAAGACCTTCGCCCCTGAAGAGGTCAGTTCCATGGTG
91     V  V  D  D  C  G  K  P  K  I  Q  V  M  Y  K  G  E  K  K  T  F  A  P  E  E  V  S  S  M  V
361   TTGACGAAGATGAAAGAAACCGCCGAGGCTTATTTGGGGTCTCCTGTGAAAGATGCAGTCGTTACGGTCCCTGCGTACTTCAATGATTCC
121    L  T  K  M  K  E  T  A  E  A  Y  L  G  S  P  V  K  D  A  V  V  T  V  P  A  Y  F  N  D  S
451   CAGAGACAAGCTACAAAAGATGCAGGTGTGATAGCAGGTCTAAACGTTCTTAGAATAATCAATGAACCAACAGCGGCAGCTCTGGCTTAC
151    Q  R  Q  A  T  K  D  A  G  V  I  A  G  L  N  V  L  R  I  I  N  E  P  T  A  A  A  L  A  Y
541   GGTTTGGACAAAAACTTGAAGGGCGAGAGGAATGTGCTTATCTTCGACTTGGGTGGTGGTACATTCGATGTGTCTATACTTACGATAGAC
181    G  L  D  K  N  L  K  G  E  R  N  V  L  I  F  D  L  G  G  G  T  F  D  V  S  I  L  T  I  D
631   GAAGGGTCTTTGTTTGAAGTAAGATCGACAGCAGGCGACACACATCTCGGGGGAGAGGACTTTGACAATCGGTTAGTTAATCACCTTGCT
211    E  G  S  L  F  E  V  R  S  T  A  G  D  T  H  L  G  G  E  D  F  D  N  R  L  V  N  H  L  A
721   GATGAATTCAAGCGTAAATACAAGAAAGACCTCCGAAGCAACCCCAGGGCTCTAAGACGACTGAGAACTGCCGCTGAGAGGGCTAAGAGA
241    D  E  F  K  R  K  Y  K  K  D  L  R  S  N  P  R  A  L  R  R  L  R  T  A  A  E  R  A  K  R
811   ACGCTCTCCTCGAGCACCGAAGCCAGCATCGAGATAGATGCTTTATACGAGGGTATCGATTTCTACACCAAAGTTAGCAGAGCGAGGTTC
271    T  L  S  S  S  T  E  A  S  I  E  I  D  A  L  Y  E  G  I  D  F  Y  T  K  V  S  R  A  R  F
901   GAAGAATTGTGCTCTGATCTATTCAGAGGAACTCTACAACCCGTAGAAAAAGCTCTGATGGACGCCAAGATGGACAAAGGCCAAATACAC
301    E  E  L  C  S  D  L  F  R  G  T  L  Q  P  V  E  K  A  L  M  D  A  K  M  D  K  G  Q  I  H
991   GACGTGGTGCTTGTGGGCGGCTCCACTAGAATCCCGAAGATCCAACAACTCCTCCAGCATTACTTTAACGGAAAACCGTTGAACTTGTCC
331    D  V  V  L  V  G  G  S  T  R  I  P  K  I  Q  Q  L  L  Q  H  Y  F  N  G  K  P  L  N  L  S
1081  ATCAACCCGGACGAAGCTGTAGCCTATGGTGCCGCAGTCCAAGCCGCCGTACTTACCGGAGAAACTGATTCTAAAATCCAAGACGTCTTA
361    I  N  P  D  E  A  V  A  Y  G  A  A  V  Q  A  A  V  L  T  G  E  T  D  S  K  I  Q  D  V  L
1171  CTGGTTGACGTAACTCCCTTGTCTTTGGGTATTGAAACCGCAGGTGGCGTCATGACGAAGATCATTGAACGTAACGCGAGGATTCCCTGC
391    L  V  D  V  T  P  L  S  L  G  I  E  T  A  G  G  V  M  T  K  I  I  E  R  N  A  R  I  P  C
1261  AAGCAAACTCAAGTCTTCACGACGTACGCCGACAACCAACCGGCGGTTACCGTGCAGGTATTCGAGGGTGAGAGGGCTATGACGAAAGAT
421    K  Q  T  Q  V  F  T  T  Y  A  D  N  Q  P  A  V  T  V  Q  V  F  E  G  E  R  A  M  T  K  D
1351  AATAACTTGCTTGGAACGTTCGATCTCACCGGAATACCCTTGGCTCCTAGAGGGGTTCCCAAGATAGAAGTGACCTTCGATCTAGACGCT
451    N  N  L  L  G  T  F  D  L  T  G  I  P  L  A  P  R  G  V  P  K  I  E  V  T  F  D  L  D  A
1441  AACGGAATTCTCAACGTATCTGCTAAAGACACCGGCTCCGGAAAGAGTACTAACATCACCATCAAGAACAATAAAGGCAGATTATCACAG
481    N  G  I  L  N  V  S  A  K  D  T  G  S  G  K  S  T  N  I  T  I  K  N  N  K  G  R  L  S  Q
1531  CAGGATATAGACAGAATGGTGTTCGAGGCGGAGAGGTACAAGGAGGAAGACGAACGACAAAGGGAAAGAATCTCGGCCCGTAATCAGCTG
511    Q  D  I  D  R  M  V  F  E  A  E  R  Y  K  E  E  D  E  R  Q  R  E  R  I  S  A  R  N  Q  L
1621  GAGGGCTATATCTTCCAAGTGAAGCAAGCTATTTCTGACTGTGCCGACAAGCTTAGTGCGGAAGACAAAGCTAAAATCGAGAGTGAATGT
541    E  G  Y  I  F  Q  V  K  Q  A  I  S  D  C  A  D  K  L  S  A  E  D  K  A  K  I  E  S  E  C
1711  GACGACTGCCTGAGATGGCTGGACGGCAACTCGTTGGCCGAGAAGGAAGAGTACGAAGAGAAGCGGAAGCACTTGACGAGTGTCTGCAGT
571    D  D  C  L  R  W  L  D  G  N  S  L  A  E  K  E  E  Y  E  E  K  R  K  H  L  T  S  V  C  S
1801  CCTATTATGGCCAAGTTGTACCAGAACGGGCCTCAGAACGGTCAATTTGGAAGTGGTACCGCTAGTGGAATGCCTGGTGGAAGTTGCGGC
601    P  I  M  A  K  L  Y  Q  N  G  P  Q  N  G  Q  F  G  S  G  T  A  S  G  M  P  G  G  S  C  G
1891  CAGCAAGCGGGAGGATTTACAGGTGGTCATAGTGGGCCAACGATAGAAGAAGTAGACTAA
631    Q  Q  A  G  G  F  T  G  G  H  S  G  P  T  I  E  E  V  D  *
```

C

```
1     ATGCCTGAAGAAAACCAAAATGGTGAAGTTGAAACCTTTGCCTTCCAGGCAGAAATTGCCCAGTTGATGAGTTTGATCATCAACACTTTC
1      M  P  E  E  N  Q  N  G  E  V  E  T  F  A  F  Q  A  E  I  A  Q  L  M  S  L  I  I  N  T  F
91    TACTCGAACAAGGAAATTTTCCTGCGAGAACTGATTTCCAACTCTTCAGATGCTCTGGATAAAATCCGTTACCAGTCGCTCACAAATCCA
31     Y  S  N  K  E  I  F  L  R  E  L  I  S  N  S  S  D  A  L  D  K  I  R  Y  Q  S  L  T  N  P
181   TCATGTTTGGACAGCGGCAAGGATCTTCATATTAAAATCATCCCAAACAAAAATGAGGGCACACTTACCATTATTGATACTGGTATTGGT
61     S  C  L  D  S  G  K  D  L  H  I  K  I  I  P  N  K  N  E  G  T  L  T  I  I  D  T  G  I  G
271   ATGACTAAGGCAGATCTGGTCAACAACTTGGGTACCATCGCAAAATCTGGAACTAAAGCTTTTATGGAAGCGTTGCAAGCAGGCGCAGAT
91     M  T  K  A  D  L  V  N  N  L  G  T  I  A  K  S  G  T  K  A  F  M  E  A  L  Q  A  G  A  D
361   ATCAGTATGATTGGTCAGTTTGGTGTAGGCTTCTACTCTGCTTACTTGATTGCCGATCGAGTCACGGTAATCTCTAAAAACAATGATGAC
121    I  S  M  I  G  Q  F  G  V  G  F  Y  S  A  Y  L  I  A  D  R  V  T  V  I  S  K  N  N  D  D
451   GAGCAGTACATCTGGGAGTCTTCTGCTGGCGGAAGCTTCACCATCCGCCCTGATCATGGAGAACCACTGGGTCGTGGTACAAAAATCCTT
151    E  Q  Y  I  W  E  S  S  A  G  G  S  F  T  I  R  P  D  H  G  E  P  L  G  R  G  T  K  I  L
541   CTACACATCAAGGAAGATCAAACTGAATTCCTAGAAGAAAATAAGATTAAGGAAATCGTTAAGAAACATTCGCAGTTTATCGGATACCCA
181    L  H  I  K  E  D  Q  T  E  F  L  E  E  N  K  I  K  E  I  V  K  K  H  S  Q  F  I  G  Y  P
631   ATTAAACTCTTGGTAGAGAAAGAGAGGGAAAAGGAACTGAGCGATGATGAGGCTGAAGAGGAAAAGAAGGATGAGGAGGACACCGATAAA
211    I  K  L  L  V  E  K  E  R  E  K  E  L  S  D  D  E  A  E  E  E  K  K  D  E  E  D  T  D  K
721   CCAAAAATTGAGGATGTAGGAGAAGATGAAGACGAGGACAAGAAAGACGAAAAGAAAAAGAAGAAGAAGACCATTAAAGAGAAGTACACT
241    P  K  I  E  D  V  G  E  D  E  D  E  D  K  K  D  E  K  K  K  K  K  T  I  K  E  K  Y  T
811   GAAGACGAAGAACTAAATAAGACGAAACCCATCTGGACCAGAAATCCAGACGATATCAGTCAAGAAGAATATGGTGAATTTTACAAATCG
271    E  D  E  E  L  N  K  T  K  P  I  W  T  R  N  P  D  D  I  S  Q  E  E  Y  G  E  F  Y  K  S
901   CTCACCAATGACTGGGAAGATCATCTGGCTGTAAAACATTTCAGCGTAGAAGGTCAGCTGGAATTCCGTGCTCTTCTGTTTGTACCACGT
301    L  T  N  D  W  E  D  H  L  A  V  K  H  F  S  V  E  G  Q  L  E  F  R  A  L  L  F  V  P  R
991   CGTGTGCCATTCGATCTCTTCGAAAACAAGAAACGCAAAAATAACGTCAAATTGTATGTACGCCGTGTATTCATTATGGACAACTGCGAA
331    R  V  P  F  D  L  F  E  N  K  K  R  K  N  N  V  K  L  Y  V  R  R  V  F  I  M  D  N  C  E
1081  GACATCATCCCAGAATACCTCAACTTCATTAAGGGTGTTGTAGATTCCGAAGATTTGCCTCTAAACATTTCTCGTGAAATGTTGCAACAG
361    D  I  I  P  E  Y  L  N  F  I  K  G  V  V  D  S  E  D  L  P  L  N  I  S  R  E  M  L  Q  Q
1171  AACAAGATCCTCAAAGTCATCCGTAAGAACTTAGTCAAGAAATGTTTGGAACTCTTCGAAGAATTGTCAGATGATAAGGATGGCTACAAG
391    N  K  I  L  K  V  I  R  K  N  L  V  K  K  C  L  E  L  F  E  E  L  S  D  D  K  D  G  Y  K
1261  AAGTTCTATGAACAGTTCTCGAAGAACCTCAAGTTGGGTATCCATGAAGACTCGCAGAACAGGGCCAAATTGGCAGATTTACTACGCTAC
421    K  F  Y  E  Q  F  S  K  N  L  K  L  G  I  H  E  D  S  Q  N  R  A  K  L  A  D  L  L  R  Y
1351  CACACTTCTGCCAGTGGAGACGAAGCCTGCTCGCTCAAGGATTATGTCAGCCGCATGAAGGAGAACCAGAAGCACGTCTACTATATCACG
451    H  T  S  A  S  G  D  E  A  C  S  L  K  D  Y  V  S  R  M  K  E  N  Q  K  H  V  Y  Y  I  T
1441  GGCGAAAGCAAAGAACAAGTAGCGCACTCGGCCTTTGTCGAACGTGTCAAGAAACGTGGCTTCGAGGTAGTCTACATGACCGAACCCATC
481    G  E  S  K  E  Q  V  A  H  S  A  F  V  E  R  V  K  K  R  G  F  E  V  V  Y  M  T  E  P  I
1531  GACGAGTATGTTGTTCAACAGCTAAAGGAATACGACGGTAAATCCTTAGTTTCCGTAACCAAAGAAGGTTTGGAGTTGCCGGAAGATGAT
511    D  E  Y  V  V  Q  Q  L  K  E  Y  D  G  K  S  L  V  S  V  T  K  E  G  L  E  L  P  E  D  D
1621  GAAGAAAAGAAGAAACGTGAGGAGGATAAGGCCAAATTTGAAGGCCTGTGCAAAGTCATGAAGACCATTTTGGACAATAAGGTGGAAAAA
541    E  E  K  K  K  R  E  E  D  K  A  K  F  E  G  L  C  K  V  M  K  T  I  L  D  N  K  V  E  K
1711  GTAGTTGTATCCAATCGTCTAGTCGAATCTCCATGTTGCATCGTAACATCCCAATATGGTTGGTCGGCCAACATGGAACGTATCATGAAA
571    V  V  V  S  N  R  L  V  E  S  P  C  C  I  V  T  S  Q  Y  G  W  S  A  N  M  E  R  I  M  K
1801  GCTCAGGCCCTTCGTGACACTTCCACCATGGGTTACATGGCTGCCAAGAAACATCTCGAAATCAATCCAGATCATCCTATCGTTGAGAAT
601    A  Q  A  L  R  D  T  S  T  M  G  Y  M  A  A  K  K  H  L  E  I  N  P  D  H  P  I  V  E  N
1891  CTACGTCAGAAAGCCGAAGCTGATAAAAACGATAAGGCTGTCAAAGATTTGGTTATTCTCCTCTTTGAAACCGCTCTACTTAGCTCTGGA
631    L  R  Q  K  A  E  A  D  K  N  D  K  A  V  K  D  L  V  I  L  L  F  E  T  A  L  L  S  S  G
1981  TTCACCCTAGACGAACCTCAAGTCCATGCTTCTAGGATCTACAGGATGATTAAGCTGGGTCTGGGTATTGACGAGGAAGAAGCTATGGTT
661    F  T  L  D  E  P  Q  V  H  A  S  R  I  Y  R  M  I  K  L  G  L  G  I  D  E  E  E  A  M  V
2071  ACGGATGAAGCTCCAGCAGGGGATGCCCCCACAGCAGAGGCTGGTGATGCTGAGGATGCTTCCCGCATGGAAGAAGTTGATTAA
691    T  D  E  A  P  A  G  D  A  P  T  A  E  A  G  D  A  E  D  A  S  R  M  E  E  V  D  *
```

D

```
1     ATGAAGCAGTACATATTTTTATTCATAGGTTTATTTATTCTTATAGGTACCACACAAGCGAGTGAAGCTGGGGGATCAGAAAATGTTGAA
1      M  K  Q  Y  I  F  L  F  I  G  L  F  I  L  I  G  T  T  Q  A  S  E  A  G  G  S  E  N  V  E
91    ACTGTTGATATAGACTTGGGGGCCAGCAGGGAAGGTTCAAGAACAGATGACGAAGTTGTTAAACGTGAAGAGGAGGCAATTAAGCTCGAT
31     T  V  D  I  D  L  G  A  S  R  E  G  S  R  T  D  D  E  V  V  K  R  E  E  E  A  I  K  L  D
181   GGCCTCAATGTGGCTCAGCTTAAGGAGCTTCGTGAGAAATCTGAGAAATTCGCATTCCAGACCGAAGTTAACAGGATGATGAAACTCATT
61     G  L  N  V  A  Q  L  K  E  L  R  E  K  S  E  K  F  A  F  Q  T  E  V  N  R  M  M  K  L  I
271   ATTAATTCTCTTTATAGGAATAAAGAAATCTTCTTGAGGGAATTGATCTCAAATGCCTCCGACGCTTTGGATAAGATCCGTATGTTATCT
91     I  N  S  L  Y  R  N  K  E  I  F  L  R  E  L  I  S  N  A  S  D  A  L  D  K  I  R  M  L  S
361   CTCACGGACAGAAGTGTTCTGGATGTTCAACCAGAGCTGAACATTCGAATAAAAGCGGATAAAGACTCGGGAATGCTGCATATCACGGAC
121    L  T  D  R  S  V  L  D  V  Q  P  E  L  N  I  R  I  K  A  D  K  D  S  G  M  L  H  I  T  D
451   ACAGGCATAGGTATGACGAAACAGGACCTTGTCAACAACCTGGGTACTATAGCAAAGTCTGGAACTGCCGAGTTCCTCAGTAAGATGCAG
151    T  G  I  G  M  T  K  Q  D  L  V  N  N  L  G  T  I  A  K  S  G  T  A  E  F  L  S  K  M  Q
541   AATGCGGAGTCGTCTCAAGATATGAATGATATGATCGGACAGTTTGGTGTTGGTTTCTACTCTGCCTTCTTGGTAGCCGATAAGGTCATT
181    N  A  E  S  S  Q  D  M  N  D  M  I  G  Q  F  G  V  G  F  Y  S  A  F  L  V  A  D  K  V  I
631   GTCACATCTAAGCACAACGATGATAAACAGTATATTTGGGAGTCTGACTCGGCAACCTTCAGTATCGTGGAAGATCCTAGAGGTGACAGT
211    V  T  S  K  H  N  D  D  K  Q  Y  I  W  E  S  D  S  A  T  F  S  I  V  E  D  P  R  G  D  S
721   TTGAAGAGAGGTACCACTGTGAGTTTGGAACTGAAACCAGAGGCCAAGGATTTCTTGGAACACGATACTGTCCGCACCTTAGTCAAGAAA
241    L  K  R  G  T  T  V  S  L  E  L  K  P  E  A  K  D  F  L  E  H  D  T  V  R  T  L  V  K  K
811   TACTCACAGTTTATTAACTTCCCGATTTATCTGTGGACCAGTCACACGGAAACTGTAGAGGAACCAGTTGAAGAGGAAGTAGCAGAGGAA
271    Y  S  Q  F  I  N  F  P  I  Y  L  W  T  S  H  T  E  T  V  E  E  P  V  E  E  E  V  A  E  E
901   AAACCGGAAACTGCCGATGAAGAAGATGCTGCTGTCGAGGAAGAGAAAGAGGACGAGAAACCAAAGACCAAGAAGGTTGAGAAGACGGTT
301    K  P  E  T  A  D  E  E  D  A  A  V  E  E  E  K  E  D  E  K  P  K  T  K  K  V  E  K  T  V
991   TGGGATTGGGAGCTTCTGAATGACAGCAAGCCGATTTGGACTAGGAAGCCTTCAGAAATCGAAGATAAGGAGTACGACGAATTCTACAAA
331    W  D  W  E  L  L  N  D  S  K  P  I  W  T  R  K  P  S  E  I  E  D  K  E  Y  D  E  F  Y  K
1081  GCCCTAACCAAGGACACCAATTCGCCTTTGACCAAAATTCATTTTGTAGCCGAGGGCGAAGTCACTTTCAAGGCCTTATTGTACGTGCCC
361    A  L  T  K  D  T  N  S  P  L  T  K  I  H  F  V  A  E  G  E  V  T  F  K  A  L  L  Y  V  P
1171  ACCGTCCAACCGTCTGAGAGCTTCAACAAATATGGCACCAAAACAGACAATATAAAATTATATGTTCGTCGCGTATTCATCACTGACGAA
391    T  V  Q  P  S  E  S  F  N  K  Y  G  T  K  T  D  N  I  K  L  Y  V  R  R  V  F  I  T  D  E
1261  TTTAACGACATGATGCCTTCATACTTGAACTTTGTCAGAGGAGTTGTGGACTCGGACGACCTACCTTTGAACGTATCTCGTGAAACCTTG
421    F  N  D  M  M  P  S  Y  L  N  F  V  R  G  V  V  D  S  D  D  L  P  L  N  V  S  R  E  T  L
1351  CAACAACACAAACTTATCAAGGTGATCAAGAAAAAGTTGGTCCGTAAAGTACTGGACATGCTCAAGAAGCTTCCTGAGGAGGACTTTGAG
451    Q  Q  H  K  L  I  K  V  I  K  K  K  L  V  R  K  V  L  D  M  L  K  K  L  P  E  E  D  F  E
1441  AAGTTTTGGAAGGAATTCTCCACAAACATCAAGCTGGGAGTTATCGAGGATCCAACAAATCGCTCCAGATTGGCCAAACTTCTTCATTTC
481    K  F  W  K  E  F  S  T  N  I  K  L  G  V  I  E  D  P  T  N  R  S  R  L  A  K  L  L  H  F
1531  TTCTCCTCCAATGACAATAAACAGACTGGCTTAGCAGAATATGTGAGCCGGATGAAGCCCAAACAAGACAAAATCTTCTACATCGCAGGA
511    F  S  S  N  D  N  K  Q  T  G  L  A  E  Y  V  S  R  M  K  P  K  Q  D  K  I  F  Y  I  A  G
1621  GCCTCGAAAGCCGAGGTGCAAAAGTCACCTTTTGTTGAACGGCTGTTGCGTAAGGGATACGAAGTCTTGTACCTGGTAGAAGCGGTCGAT
541    A  S  K  A  E  V  Q  K  S  P  F  V  E  R  L  L  R  K  G  Y  E  V  L  Y  L  V  E  A  V  D
1711  GAATATACTATTTCTGCCCTTCCTGAATTTGAAGGCAAGAAATTCCAGAACGTTGCCAAGGAAGGGTTCTCCTTAACCGAAAGTGAAGGA
571    E  Y  T  I  S  A  L  P  E  F  E  G  K  K  F  Q  N  V  A  K  E  G  F  S  L  T  E  S  E  G
1801  GGCAAGGACCAATTGGATCAGTTGAAGACCACTTTTGAGCCCCTCACCAAATGGTTGTCGGAAAACGCTCTGAAGGATCACGTTGCCAAA
601    G  K  D  Q  L  D  Q  L  K  T  T  F  E  P  L  T  K  W  L  S  E  N  A  L  K  D  H  V  A  K
1891  GCTATGGTATCCGAAAGATTGTCAGATTCCCCTTGTGCTCTAGTAGCAAGCTTGTTCGGCTGGACCGGAAACATGGAAAGACTTGCCGTT
631    A  M  V  S  E  R  L  S  D  S  P  C  A  L  V  A  S  L  F  G  W  T  G  N  M  E  R  L  A  V
1981  TCCAATGCCCATCAAAAATCAGATGATCCTCAGAGGTCCTACTATTTGAACCAAAAGAAGACCTTGGAGATCAATCCAAGACACCCGCTG
661    S  N  A  H  Q  K  S  D  D  P  Q  R  S  Y  Y  L  N  Q  K  K  T  L  E  I  N  P  R  H  P  L
2071  ATGCGAGAGTTATTGAAGAGGGTTAACGATGATGCGGATGACCCGACAGCGAAAGACATGGCACTGATGATGTTCAGAACGGCAACTCTG
691    M  R  E  L  L  K  R  V  N  D  D  A  D  D  P  T  A  K  D  M  A  L  M  M  F  R  T  A  T  L
2161  AGATCTGGGTACATGCTGAAAGATACATCCGATTTTGCCCAGTCCATCGAAGTTATGATGAGAAAGACGCTAGGGGTTCCTCTGGACGAG
721    R  S  G  Y  M  L  K  D  T  S  D  F  A  Q  S  I  E  V  M  M  R  K  T  L  G  V  P  L  D  E
2251  CAAGTGGAGGAAGAGGAAGATGTTCCCGAGGATGGCATTCCAGAGGACGAGAATGGAGATATTAAGGATGAGGAGGAGAGAGACGAAGAG
751    Q  V  E  E  E  E  D  V  P  E  D  G  I  P  E  D  E  N  G  D  I  K  D  E  E  E  R  D  E  E
2341  CATGACGAACTGTAA
781    H  D  E  L  *
```

Figure S1 The coding sequences and their deduced amino acid sequences for AglaHsp20.43, AglaHsp71.18, AglaHsp82.09, and AglaHsp89.76. The signal peptide sites and protein conserved domains are marked with black and red underscores, respectively. The signature sequences and subcellular localization motifs are marked with gray and red backgrounds, respectively.
