# Supplementary material for: Cloning and Molecular Characterization of Hsp Genes from Anoplophora glabripennis and Their Responses to Cold Stress
Source: Int J Mol Sci. 2022 Oct 8;23(19):11958. doi: 10.3390/ijms231911958 (PMC9570420; doi:10.3390/ijms231911958)
Supplement: Supplementary file 1 [file ijms-23-11958-s001.zip › Figure S2 Expression profiles of AglaHsp genes under different low temperature treatments for 6 h.pdf]

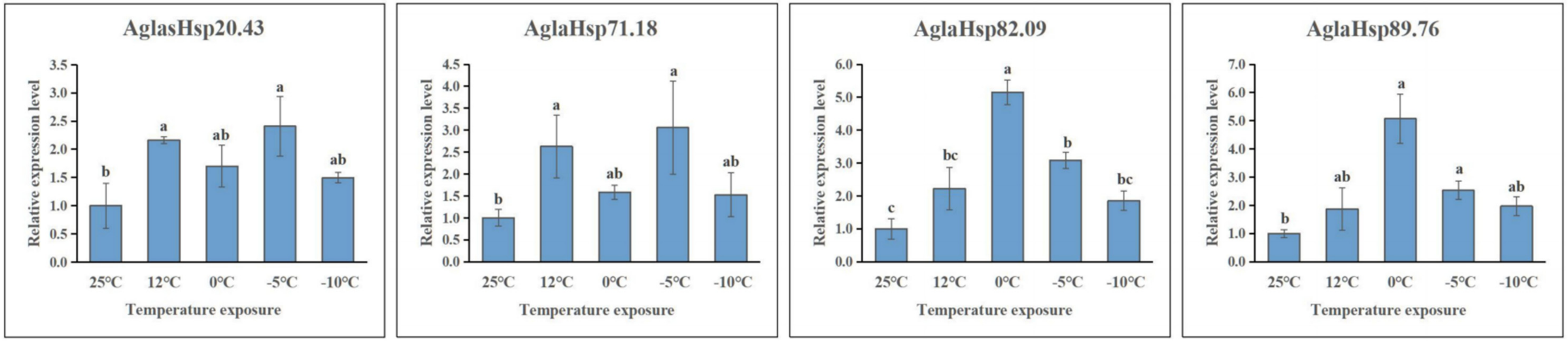

Figure S2 Expression profiles of *AglaHsp* genes under different low temperature treatments for 6 h. Gene expression at 25°C was used as control, and relative expression levels are expressed as means ± standard error of mean (SEM). Lowercase letters above the error bar indicate significant differences ($P < 0.05$, LSD
